# Diphtheria

**Published:** 1874-12

**Authors:** 


### DIPHTHERIA

Has two prominent characteristics, a very bad breath and great bodily debility or prostration, with some discomfort about the throat, and whitish patches, all caused by want of cleanliness, of good ventilation, by dampness, and by colds. There is no sufficient reason to believe that it is catching by ordinary association in the same room, except so far as the same surroundings which occasioned the disease in the patient would cause it in another person exposed to like influences, unless the other person were in rigorous health, and able to repel the tendency to the malady. The acid tannate of iron, applied to the diseased parts inside the mouth and palate with a brush, is believed to be the best astringent remedy known up to this time; but, as the substance is not readily had, the diseased part may be painted with a brush dipped in muriated tincture of iron, and then, in five minutes or less, a strong solution of tannin may be applied. In a few hours the discolored appearance will show the extent of the disease, and separation will go on rapidly, if the system is sustained by nourishing food.

